# Nitrate-Selective Anion Exchange Membranes Prepared using Discarded Reverse Osmosis Membranes as Support

**DOI:** 10.3390/membranes10120377

**Published:** 2020-11-27

**Authors:** Amaia Lejarazu-Larrañaga, Juan Manuel Ortiz, Serena Molina, Yan Zhao, Eloy García-Calvo

**Affiliations:** 1 IMDEA Water Institute, Avenida Punto Com, 2, 28805 Alcalá de Henares, Madrid, Spain; juanma.ortiz@imdea.org (J.M.O.); serena.molina@imdea.org (S.M.); eloy.garcia@imdea.org (E.G.-C.); 2 Chemical Engineering Department, University of Alcalá, Ctra. Madrid-Barcelona Km 33.600, 28871 Alcalá de Henares, Madrid, Spain; 3 Department of Chemical Engineering, Katholieke Universiteit of Leuven (KU Leuven), Celestijnenlaan 200F, B-3001 Leuven, Belgium; yan.zhao@kuleuven.be

**Keywords:** selective separation, heterogeneous anion exchange membrane, membrane recycling, nitrate separation, transport numbers

## Abstract

The present work shows a methodology for the preparation of membranes with a high affinity for nitrates. For this purpose, a polymeric mixture containing an anion exchange resin was extended on a recycled pressure filtration membrane used as mechanical support. Different ion exchange resins were tested. The influence in ion fractionation of (i) the type of ion exchange resin, (ii) the use of a recycled membrane as support and (iii) the operating current density during the separation process were studied. Results revealed that the employed anion exchange resin could tune up the transport numbers of the anions in the membrane and enhance the transport of nitrates over sulfates. The use of the recycled filtration membrane as support further increased the transport of nitrates in detriment of sulfates in nitrate-selective membranes. Moreover, it considerably improved the mechanical stability of the membranes. Lowering the operational current density also boosted ion fractionation. In addition, the use of recycled membranes as support in membrane preparation is presented as an alternative management route of discarded reverse osmosis membranes, coupling with the challenging management of waste generated by the desalination industry. These membranes could be used for nitrate recovery from wastewater or for nitrate separation from groundwater.

## 1. Introduction

Nitrate pollution in natural waters has become a worldwide issue, mainly caused by the abusive use of chemical fertilizers and insufficient wastewater treatment [1]. The excessive concentration of nitrates in surface and groundwater causes the eutrophication of rivers and lakes and the pollution of drinking water. In addition, nitrate is toxic to human health, causing methemoglobinemia and associated with cancer and adverse reproductive outcomes [2]. Thus, the European Commission limits nitrate concentration in drinking water to a maximum level of 50 ppm but recommends to keep it below 25 ppm [3,4]. This situation has motivated increasing research in the development of effective technologies for nitrate removal [5].

Commonly used technologies for nitrate removal include ion exchange resins, membrane technology reverse osmosis (RO), ion exchange membranes (IEMs) or biological and chemical treatments. Among them, membrane technology could be more appropriate to avoid secondary contamination problems in the treated water, which are commonly caused by the use of chemical products and microorganisms [5]. In contrast to RO, where all the dissolved compounds are removed from the feed, special grade IEMs could selectively separate nitrates from a multi-component solution.

IEMs are electrically charged selective barriers with the ability to repulse co-ions (ions with the same charge as the fixed ionic groups in the membrane) while allowing the permeation of counter-ions (oppositely charged ions). This phenomenon is known as the Donnan-exclusion effect, and it is expressed in terms of membrane permselectivity [6]. However, conventional IEMs have a poor ability to discriminate between the counter-ions. The preparation of IEMs with selectivity to specific counter-ions has been intensively researched since the 1950s [6,7]. Despite this, the achievement of membranes with high permselectivity to specific ions together with other desired membrane properties (low cost of production, chemically and mechanically stable, low electrical resistance) is still challenging to date [8,9].

There are several mechanisms for achieving selectivity to specific counter-ions in IEMs, roughly based on (i) sieving effect, (ii) enhanced Donnan-exclusion effect, and (iii) specific interactions between the ions in the solution and the ionic sites in the membrane [10]. First, the sieving effect can be enhanced by narrowing the ion channel paths and obtaining dense membrane structures, for instance, by increasing the cross-linking of the polymers in the membrane matrix [11,12] or by the deposition of a highly cross-linked layer on the membrane surface [13]. Second, the Donnan-exclusion effect against multivalent ions can be increased by the deposition of an oppositely charged layer on the membrane surface. There are several techniques for surface modification, including adsorption [14,15], coating [16] and layer-by-layer deposition of charged materials [17,18]. Third, specific interactions between the membrane and the ions in the solution can promote a selective separation, such as host–guest molecular interactions [19] or hydrophobic interactions [20,21,22].

A huge research effort has been devoted to the modification of commercially available IEMs with advanced materials for enhancing selective properties, including graphene, carbon nanotubes and conductive nanoparticles [23,24,25]. However, the use of these advanced materials could be far away from a reasonable cost-efficiency of the industrial production and application of these membranes [26]. Another strategy for improving the transport of ions with lower charge is lowering the current density during the separation process. When the current density is relatively high, the concentration of counter-ions in the membrane increases and could saturate the fixed charges in the membrane, decreasing the Donnan exclusion. This can be reflected in an indiscriminate exchange of ions, including counter- and co-ions. On the contrary, when the applied current density is relatively low, ions with high apparent activation energy (multivalent) may have an insufficient electric charge to face the energetic barrier to be transported, enhancing the separation between monovalent and multivalent ions [27,28,29]. Few research works have been devoted to the introduction of pressure filtration membranes (i.e., nanofiltration (NF) or ultrafiltration (UF) membranes) in the ED stack, alternated with IEMs, for increased separation of multivalent ions due to a sieving effect. The fractionation of ions was significantly enhanced by the insertion of NF membranes alternated with IEMs, though the electrical resistance of the stack was considerably raised due to the non-conductive nature of the NF membranes [30,31,32].

Based on the aforementioned previous research, in the current work, we have combined different strategies for increasing the selectivity to nitrates. First, we employed different types of anion exchange resins for membrane preparation. These resins differed in the length of the alkyl chains bonded as substituents to the quaternary amine, which promoted differences in the hydrophobic interactions of the resulting membranes. These hydrophobic interactions could enhance the repulsion of highly hydrated ions (i.e., sulfates) while promoting the permeation of ions with low water solvation molecules (i.e., nitrates). Second, we have tried to boost the sieving effect by using a recycled pressure filtration membrane with ultrafiltration properties as mechanical support. For that purpose, we cast a polymeric solution containing an anion exchange resin directly on the surface of a recycled ultrafiltration-like membrane. By means of this, we expected an enhanced sieving effect due to the combination of the recycled ultrafiltration support and the dense and charged polymeric casting. In addition, the membranes employed as mechanical support have been obtained from an end-of-life reverse osmosis (RO) module that was discarded by a desalination plant [33,34]. In this way, we attempt to boost the sustainability in membrane technology by presenting an alternative management route for the end-of-life membranes that regularly end up in landfills (>840,000 RO end-of-life membranes discarded every year, >14,000 tons of plastic waste and expected to keep growing [35,36]). Finally, we have tested the membranes under different current densities in order to evaluate their influence on ion fractionation. Ternary mixtures of sodium salts containing an equimolar concentration of anions (NO_3_^−^, Cl^−^ and SO_4_^2−^) were used to compare the flux of each anion through the membranes. Results show that the type of anion exchanger could constitute the main parameter affecting the selective separation of anions.

## 2. Materials and Methods

### 2.1. Chemical Reagents

Sodium hypochlorite (NaClO, 14%), tetrahydrofuran (THF), sodium chloride (NaCl), sodium sulfate (Na_2_SO_4_) and sodium nitrate (NaNO_3_) were purchased from Scharlab S.L., Barcelona, Spain Polyvinylchloride (PVC, Mw = 112,000 g·mol^−1^) was supplied by ATOCHEM S.A., Madrid, Spain. Amberlite^®^ IRA-402 and Lewatit^®^ Sybron Ionac^®^ SR-7 anion exchange resins were supplied by Merck KGaA, Darmstadt, Germany. Purolite^®^ A600/9413 anion exchange resin was supplied by MemBrain^®^ s.r.o., Stráž pod Ralskem, Czech Republic. The characteristics of the anion exchange resins are reported in Table 1. MilliQ water was used throughout the experiments.

### 2.2. Recycled Membrane Support and Commercial Membranes

An end-of-life (EoL) polyamide thin film composite (PA-TFC) RO membrane (TM 720–400, Toray Industries, Inc., Osaka, Japan), used and discarded by a brackish water desalination plant located in Spain, was used in this study as a mechanical support in membrane preparation [33]. For that purpose, the discarded RO module was first pretreated with NaClO in a recycling pilot plant to remove the fouling and the polyamide (PA) layer and then opened by membrane autopsy for the extraction of membrane coupons [37,38]. It was demonstrated in previous works that the employed exposure dose to the oxidizing agent (800,000 ppm h NaClO) ensures the complete elimination of the PA layer. Thus, the resulting membranes show a porous polysulfone surface and have ultrafiltration-like properties (in terms of permeability and salt rejection) [39,40]. The membranes were carefully rinsed with MilliQ water and stored in wet conditions. These membranes (named as RE-UF, henceforth) were used as a mechanical support in the preparation of the anion exchange membranes (EMs).

Ralex^®^ AMH-PES membrane from Mega a.s. (Straz pod Ralskem, Czech Republic) was used to compare the performance of the prepared membranes with a commercial one. Neosepta CMX membranes from Astom Corp. (Tokyo, Japan) were used as auxiliary cation exchange membranes (CEM), for the separation of anolyte/catholyte compartments, in electro-separation experiments.

### 2.3. Anion Exchange Membrane Preparation

The EMs were prepared by casting method as it was described before in [33,34]. The anion exchange resins were first dried and pulverized until a − 300 + 400 mesh size was ensured. The polymeric solution was prepared by dissolving PVC into THF and dispersing the finely grounded anion exchange resin in the solution. This mixture was properly stirred and sonicated to ensure a homogeneous distribution of the resin particles. The membranes were prepared by extending the polymeric mixture either on the surface of the recycled membrane (membranes named as Amb-RE-UF, Puro-RE-UF, Lew-RE-UF) or in a clean glass plate (membranes named as Amb, Puro, Lew). In all the cases, the mixture was cast with 800 µm thickness using a doctor blade, and the solvent was evaporated for 60 min at room temperature. After the solvent evaporation, membranes were immersed in a water bath at 20 °C. Finally, the membranes were rinsed with MilliQ water and stored wet. Table 2 summarizes the membranes under study.

### 2.4. Membrane Characterization

#### 2.4.1. Scanning Electron Microscopy (SEM) and Energy-Dispersive X-ray Spectroscopy (EDX)

The surface morphology of the prepared membranes was analyzed by scanning electron microscopy (SEM) using an XL30 ESEM Model (Phillips N.V., Amsterdam, the Netherlands). The membrane elemental composition was analyzed using a Bruker Nano X-ray detector by dispersive energy (EDX) and equipped with an XFlash detector 5030 coupled to a FESEM S-8000 Model (Hitachi, Ltd., Tokyo, Japan). Prior to the analysis, the samples were dried at 50 °C for 48 h. Then, all the samples were chrome sputtered with a Sputter Coater Quorum Q150T ES model (Quorum Technologies Ltd., Laughton, United Kingdom) to achieve 13–15 nm-thickness.

#### 2.4.2. Thickness, Ion Exchange Capacity, Water Content

Membrane thickness was measured by a digital Mitutoyo micrometer IP65 model (Mitutoyo Corp., Kawasaki, Japan) in the swollen state. Before the measurement, the membrane was wiped off with a filter paper.

The ion exchange capacity (IEC) evaluates the number of functional groups present per gram of dried membrane (mmol·g^−1^). For evaluating the IEC, membranes were first immersed in KNO_3_ 1 M solution and stirred for 24 h. Then, the samples were washed with MilliQ water and submerged in NaCl 0.5 M under stirring for 24 h. The concentration of the NO_3_^-^ released in the NaCl solution was measured by UV-vis spectrophotometer, UV-1800 SHIMADZU (Shimadzu Corp., Kyoto, Japan), and the *IEC* was calculated as follows [33,41]

(1) $$IEC=\;\left(\frac{n_{NO_{3}^{-}}}{W_{dry}}\right)$$

where $n_{NO_{3}^{-}}$ (mmol) is the NO_3_^−^ mol number present in the NaCl solution, and *W_dry_* (g) is the dry weight of the membrane.

The water content (*WC*) indicates the amount of water that is swelled by the membrane. It was analyzed by gravimetric method, following Equation (2):

(2) $$WC=\;\left(\frac{W_{wet}-W_{dry}}{W_{dry}}\right)\cdot 100$$

where *W_wet_* (g) is the weight of the membrane swollen in water, and *W_dry_* (g) is the weight of the membrane after been dried in an oven until constant weight.

#### 2.4.3. Electrochemical Properties

The membrane under study was placed between two compartments cell as described in [33]. The potential difference across the membrane was measured by two Ag/AgCl reference electrodes. The effective membrane surface was 4.52 cm^2^.

For measuring the electrical resistance, both compartments were filled with NaCl solution (0.5 M). Carbon felt electrodes were used as anode and cathode. The solutions were continuously stirred. An external potential was applied and increased stepwise; the potential drop in the cell was measured by the reference electrodes. The potential drop in the cell was analyzed with and without membrane, and the electrical resistance was graphically calculated using Ohm´s law (Equation (3)), as follows [42]:

(3) $$R=\frac{U}{I}$$

where *R* (Ω) is the electrical resistance, *U* (V) the potential and *I* (A) the current:

(4) $$R_{m}=\left(R_{2}-R_{1}\right)\cdot A$$

where *R*_2_ (Ω) is the electrical resistance of the cell with the membrane, *R*_1_ (Ω) is the electrical resistance of the cell without the membrane, and *A* (cm^2^) is the effective membrane surface.

Membrane permselectivity indicates its ability to repulse the co-ions under passive conditions (i.e., without the application of an external potential). For its evaluation, the membrane under study was placed in the test cell, and the compartments were filled with NaCl solutions of different concentrations (0.1 M and 0.5 M). The solutions were continuously stirred for 30 min of equilibration. After this, the potential difference across the membrane was measured with the reference electrodes. The permselectivity was calculated as follows [42]:

(5) $$\alpha =\;\frac{\Delta V_{exp}}{\Delta V_{t}}\;100\%$$

where *α* (%) is the permselectivity, *∆V_m_* is the experimental potential difference, and *∆V_t_* is the theoretical potential difference for a 100% permselective membrane. *∆V_t_* was calculated by the Nernst equation:

(6) $$E_{m}=\frac{R\cdot T}{z\cdot F}\cdot \;\left(2t_{i}-1\right)\cdot ln\frac{a_{1}}{a_{2}}$$

where *E_m_* (V) is the potential difference, *R* (J·mol^−1^·K^−1^) the gas constant, *T* (K) is the temperature, *z* is the electrovalence of the electrolyte, *F* (C·mol^−1^) is the Faraday constant, *t_i_* is the transport number for 100% permselective membrane (*t_i_* = 1), *a_1_* and *a_2_* are the activity coefficients of NaCl solutions [43].

### 2.5. Evaluation of the Selective Ion Transport Properties

**Figure 1:**
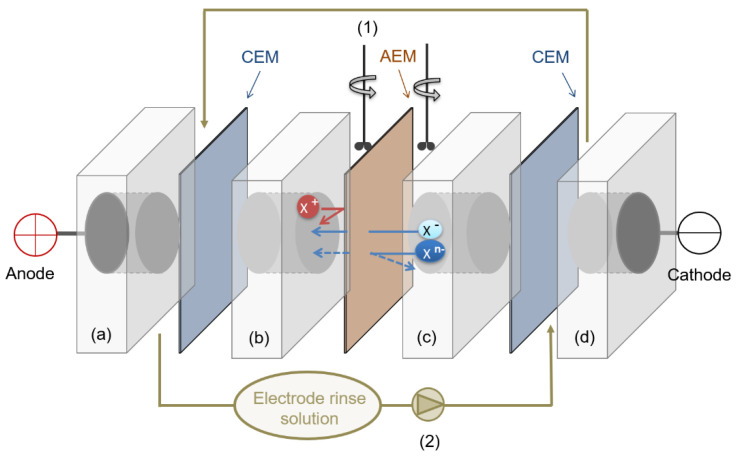
Experimental set up employed for the evaluation of membrane transport properties. CEM: cation exchange membrane (Neosepta CMX). EM: anion exchange membrane (under analysis). Electrodes: dimensional stable electrodes (ti/mixed metal oxides). Electrode compartments (**a**,**d**): 5 cm-thickness, 28.27 cm^2^ cross-sectional area, 3.6 L·h^−1^ flow rate. Concentrate (**b**) and dilute (**c**) compartments: 5 cm-thickness, 19.64 cm^2^ cross-sectional area, under stirring. (1) Mechanical rod stirrer, (2) peristaltic pump.

The evaluation of the separation capacity was performed in a four compartments test cell (see Figure 1), under active conditions (i.e., by the application of an external potential).

The effective membrane area was 19.64 cm^2^. Dimensionally stable electrodes (DSE, titanium coated with iridium oxide, provided by Inagasa S.A., Barcelona, Spain) were employed as anode and cathode. A solution of Na_2_SO_4_ 0.2 M was circulated for electrode rinse by a peristaltic pump (3.6 L·h^−1^). An equimolar mixture of monovalent and divalent anions (Cl^−^, NO_3_^−^ and SO_4_^2−^, 50 mM) added as their sodium salts was used as feed. The volume of the dilute and concentrate compartments was 0.1 L, mechanical rod stirred were used for proper mixing. The experiments were performed at constant current (CC), at two different current densities (3.5 mA·cm^−2^ and 10 mA·cm^−2^). In this way, the effect of the current density on the selective separation was evaluated for each studied membrane. All the experiments were performed at room temperature (25 °C). The overall performance of the process was controlled by conductivity measurements using a conductivimeter (PC 52+ DHS XS, from XS instruments). The concentration of each anion in the diluted compartment during the experiments was measured using an 861 advanced compact IC Metrohm ionic chromatograph. Membrane transport properties were evaluated in terms of ionic molar flux ($J_{i}^{m}$), transport numbers ($t_{i}^{m}$), permselectivity between ions or relative transport number ($P_{B}^{A}$) and separation efficiency (*S*). These terms were calculated following the Equations (8)–(11).

The total flux of ions through the membrane from the dilute to the concentrate compartment is directly related to the current density,

(7) $$j=F\;\sum \left|z_{i}\right|J_{i}^{m}$$

where *j* (mA·cm^−2^) is the current density, *F* is Faraday’s constant, *z_i_* is the electric charge of the ion and $J_{i}^{m}$ is the flux of the ion.

In this sense, the flux of each ion ($J_{i}^{m}$) could be expressed with the next equation [44]:

(8) $$J_{i}^{m}=t_{i}^{m}\frac{j}{\left|z_{i}\right|\cdot F}$$

where $t_{i}^{m}$ is the transport number of the ion in the membrane phase.

The transport number of an ion in the membrane phase ($t_{i}^{m}$), quantifies the fraction of the charge that is carried through the membrane by a specific ion during the electro separation process can be calculated as:

(9) $$t_{i}^{m}=\frac{\left|z_{i}\right|\cdot J_{i}^{m}}{\sum |z_{i}|\cdot J_{i}^{m}},$$

The permselectivity between two anions or relative transport number of A to B ($P_{B}^{A}$) (Equation (10)) indicates the ratio of charge that is transported by component A compared to component B (usually the ion with lower transport number in the membrane) divided by the ratio of concentrations (in equivalents) of both ions [45]:

(10) $$P_{B}^{A}=\frac{t_{A}/t_{B}}{C_{A}/C_{B}}$$

where *t_A_* and *t_B_* are the transport numbers of A and B ions in the membrane phase, and CA and *C_B_* (eq.·L^−1^) are the concentrations, both for components A and B. The interest of this parameter is because it is useful to predict the behavior of the studied membrane in electrodialysis separations under different experimental conditions.

If we measure in an experiment the transport numbers for A and B, *t_A_* = 0.6 and *t_B_* = 0.4, in a solution with CA = *C_B_* = 1 eq·L^−1^, and having the same electric charge *z_A_* = *z_B_* = 1, the value of the relative transport number of A to B will be $P_{B}^{A}=\;$1.5. In this simple example, it is possible to deduce that 1.5 equivalents of component A are transported through the membrane by migration for each equivalent of B that is transported.

Even if the transport number of each ion depends on its concentration in the solution, the parameter $P_{B}^{A}$ could be considered reasonably constant during the experiment (when *j < j_lim_* and using diluted concentrations). Hence, if the experiment is repeated with CA = 2 eq·L^−1^ and *C_B_* = 1 eq·L^−1^, as the value of $P_{B}^{A}$ = 1.5, it is possible to conclude that the rate $\frac{t_{A}}{t_{B}}$ = 3, indicating that 3 equivalents of component A would be transported for each equivalent of B. From a general point of view, $P_{B}^{A}$ > 1 indicates a preferential transport of component A in respect to component B. It should be avoided to mix up the concepts of permselectivity between the counter-ions A and B $\left(P_{B}^{A}\right)$ with the permselectivity between counter-ions and co-ions (*α*), reported in Equation (5).

Hence, $P_{B}^{A}$ is measured under active conditions (i.e., by the application of an external potential), and it is related to the separation efficiency between the counter-ions in a multi-component mixture (i.e., NO_3_^−^, Cl^−^, SO_4_^2−^). Though α refers to the affinity of the membrane for a reference counter-ion (i.e., Cl^−^ in EMs) in respect to a reference co-ion (i.e., Na^+^ or K^+^ in EMs), it is measured under passive conditions (i.e., without applying any external potential) and it is related to the current efficiency during the electro separation process.

Additionally, the separation efficiency of the membranes was calculated and reported as Supplementary Material. The separation efficiency was introduced by [46] as an alternative for more common parameters such as the separation factor in other membrane technologies (i.e., nanofiltration). It reflects the relative difference in transport rate (see Supplementary Materials, Equations (S1)–(S9)) and ranges from 0 (no separation) to 1 (complete separation, i.e., *C_B_ (t)* = 0; component B completely removed from the dilute fraction).

## 3. Results and Discussion

### 3.1. Membrane Characterization

#### 3.1.1. Main Characteristics of the Recycled Membrane Support

The recycled membrane support has been thoroughly characterized in previous works [39,40]. In the present work, the end-of-life RO membranes were subjected to 800,000 ppm·h NaClO. This exposition dose ensures the complete elimination of the fouling and the active polyamide layer and thus the achievement of UF-like properties in terms of rejection (colloidal and macro compounds) and water permeability (10–50 L·m^2^·h^−1^·bar^−1^) [39].

With respect to the morphology, the recycled membrane support is composed of two different polymeric layers: the polyester layer, as a mechanical reinforcement and the polysulfone layer, with a microporous structure [40]. This membrane support is relatively tight in comparison with other mechanical supports employed in IEM preparation (i.e., nonwoven polyester fabric [47]). On the one hand, the lower porosity of the recycled membrane support could promote a better permselectivity to the resulting IEMs. On the other hand, it could also limit the electrical conductivity of the membranes. Further, the membrane support is characterized to have a very high mechanical stability, inherited from the end-of-life RO membrane [33].

#### 3.1.2. Anion Exchange Membrane Morphology and Elemental Composition

**Figure 2:**
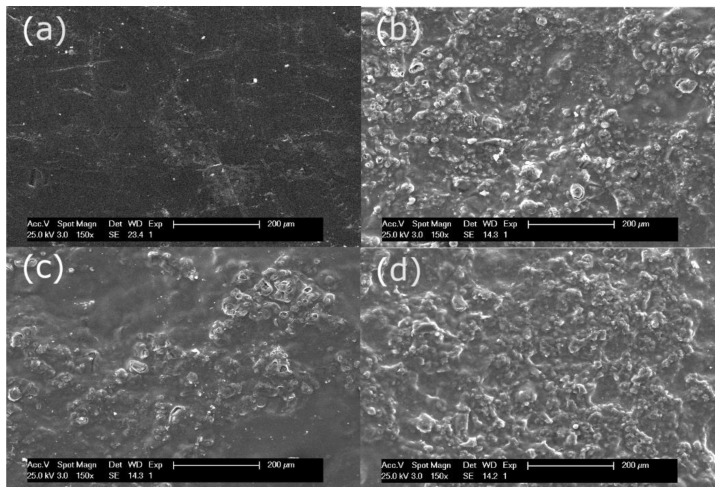
SEM surface micrographs of (**a**) Recycled ultrafiltration-like membrane (RE-UF), (**b**) Amb-RE-UF, (**c**) Puro-RE-UF and (**d**) Lew-RE-UF.

Figure 2 shows the SEM surface micrographs of RE-UF, Amb-RE-UF, Puro-RE-UF and Lew-RE-UF. Figure 2a shows the polysulfone surface of the RE-UF membrane. It can be observed that this surface is smooth and free of foulants, confirming the success of the oxidative chemical cleaning. In Figure 2b–d, it can be observed the distribution of the ion exchange resin particles on the membrane surface, bonded by the film-forming PVC. Amb-RE-UF and Lew-RE-UF membranes (Figure 2b,d) show a homogeneous distribution of the resin particles. Sonication and stirring of the polymeric mixture (before the casting) enhances the dispersion of the resin particles, reducing their agglomeration and precipitation and thus, achieving a homogeneous distribution on the membrane surface [48,49]. Differently, Puro-RE-UF (Figure 2c) shows a less uniform distribution, presenting areas with fewer ionic particles, which could be caused by a poor dispersion of the resin particles in the polymeric solution. Interactions between the Purolite anion exchanger and the polymeric mixture could cause agglomeration and precipitation of particles and the consequent nonhomogeneous distribution of the resin particles in the membrane surface. The unequal distribution of the ion exchange resin particles could cause anomalies and defects in the physicochemical and electrochemical properties of the resulting membranes [48]. Further, the Puro-RE-UF membrane presents some cracks in its surface that could promote an indiscriminate exchange of ions, reducing the permselectivity.

Additionally, EDX analysis was performed to study the chemical composition in both sides of prepared EMs (casting layer and support layer). The content of elemental measurements is shown in Table 3. The EDX images are presented in the Supplementary Materials, Figure S1. Complementarily, the EDX analysis of the RE-UF surface was performed; the elemental chemical composition is detailed in Supplementary Figure S1 and Supplementary Table S1.

The elements C, N, O, S and Cl were observed by EDX analysis on both sides of each membrane. The Cl indicated the presence of PVC, added as a binder in the casting mixture. Subsequently, Cl was not detected on the surface of the RE-UF (see Supplementary Material, Table S1). The presence of Cl in the support layer indicates that the PVC solution has penetrated the membrane support. This could be caused by the fact that the employed organic solvent (THF) has dissolved the polysulfone layer. The penetration of the PVC into the recycled support provides high adherence to the casting layer and the consequent enhancement of the mechanical properties in AEMs [33]. Still, the % atomic of Cl is higher on the casting side than on the support side. This could be owing to the fact that the PVC solution was not fully embedded in recycled support. The low % of atomic of N found in the casting layer could mean that the resin particles are almost totally covered by the PVC film. A. Awasthi et al. [50] have reported 4.24 atomic % of *N* in EDX analysis of this type of pure resins (without being embedded into a polymer matrix). Further, *N* was not detected in the support layer. It would seem reasonable to consider that the resin contained in the PVC layer is not homogeneously distributed in the entire cross-section of the polyester support. This would increase the heterogeneity in the distribution of the ionic sites across the membrane section, which could result in tortuous and noncontinuous channels for the exchange of ions. This finding is in accordance with the high resistance results found in these membranes (see Section 3.1.3).

#### 3.1.3. Thickness, Ion Exchange Capacity, Water Content

**Figure 3:**
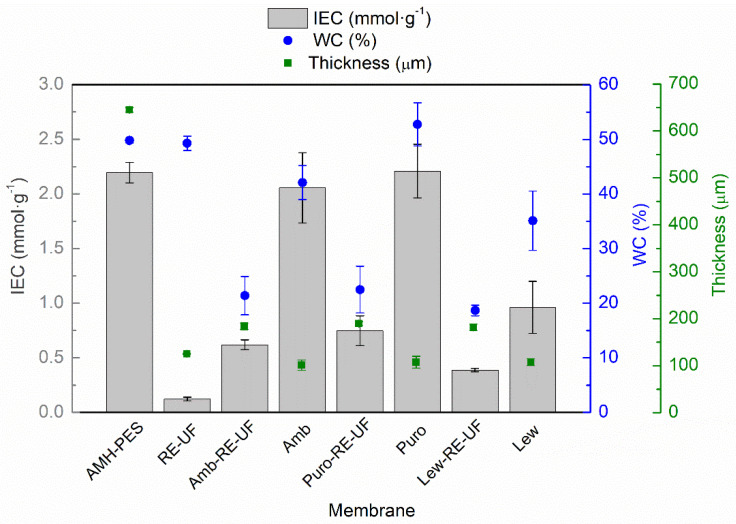
Ion exchange capacity (IEC, mmol·g^−1^); water content (WC, %) and thickness (µm) of the studied membranes.

Figure 3 reports the average thickness, water content (WC) and ion exchange capacity (IEC) of the studied membranes.

As it can be observed, the highest thickness corresponds to AMH-PES, as two layers of polyester as a support are added during production to ensure mechanical stability. The recycled membrane support (RE-UF) has an average thickness of 126 µm. After the casting, the solvent evaporation and the phase inversion, the average thickness of the EMs is around 185 µm. Interestingly, membranes without mechanical support have an average thickness of 106 µm. These measurements indicate that the polymeric solution has penetrated the mechanical support, lowering the thickness of EMs in respect to the sum of RE-UF support and membranes without support. As mentioned before, this could promote the high adherence of the casting layer to the recycled support, providing mechanical stability to the whole membrane.

The IEC of a membrane is directly related to its swelling capacity (WC) [42]. In this way, higher IEC is reflected in a greater WC, as can be observed in Figure 3. The only exception is the case of RE-UF support, which having near-zero IEC (as it is vacant of fixed functional groups), shows a high wettability. This can be attributed to the porous structure of the polysulfone in the RE-UF membrane, which increases the volume of the cavities where water can be contained [40]. On the contrary, the use of the RE-UF membrane in EM preparation reduces the WC, probably due to the formation of a compact membrane structure, where the casting is embedded on the support, reducing the cavities for containing water. Further, the EMs without mechanical support have a higher water content that could be caused by greater penetration of water in the membrane structure during the phase inversion, conforming cavities where water can be contained. Thus, the reduced IEC of the membranes with RE-UF support can be attributed to both the lower WC of these membranes and to the lowered number of functional groups per membrane mass in respect to the membranes without RE-UF. The IEC and the WC of the membranes prepared with Lewatit anion exchanger are particularly low. This could be related to the presence of propyl chains bonded to the quaternary amine (R–(C_3_H_7_)_3_N^+^), instead of the methyl groups (R–(CH_3_)_3_N^+^), that are present in Amberlite and Purolite resins (Table 1). The increase in the length of the alkyl chain bonded to the N^+^ group is related to an increased hydrophobicity of the ionic sites, which could reduce the WC and IEC of the resulting membranes [20,45]. The low WC and IEC of the EMs prepared using the recycled support could compromise the electrical conductivity of the membranes (see Section 3.1.4).

#### 3.1.4. Electrochemical Properties

**Figure 4:**
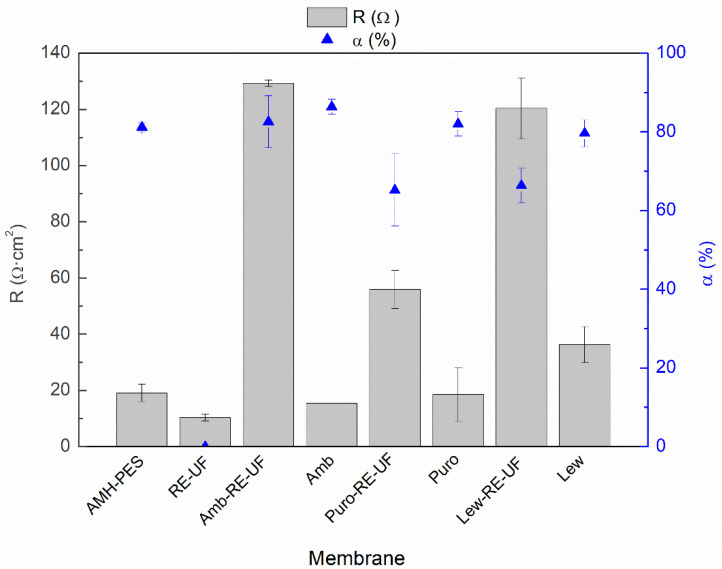
Electrical resistance (*R*, Ω·cm^2^) and permselectivity (*α*, %) of the studied membranes.

Figure 4 shows the electrical resistance (*R*) and the permselectivity (α) of the membranes.

As mentioned before, membrane properties are closely related to each other. In such a way that, high IEC and water content promote connected ion channel paths through the membrane, increasing the electrical conductivity. Moreover, excessive swelling can result in a loose structure, with too expanded ion channel paths, which can generate an indiscriminate exchange of ions and reduce the permselectivity [42,45]. Paying attention to Figure 4, it can be noticed that the electrical resistance of the prepared EM is considerably higher than the simple sum of the resistances of the casting and the RE-UF support. This effect could be attributed to the differences in the distribution of the ion exchange resin between the casting layer and the support layer, as it was demonstrated by EDX analysis (Table 3). Moreover, the formation of a compact membrane structure with a low WC and IEC could also contribute to the reduction of membrane conductivity [6,42,51]. Differently, the Puro-RE-UF membrane shows lower electric resistance than other membranes with mechanical support, which could be caused due to an accumulation of anion exchange resin particles or to the presence of cracks in the surface of the tested membranes (see Figure 2b). Membranes without the mechanical support show a relatively low electrical resistance, more similar to the commercial membrane (AMH-PES). However, without mechanical support, the membranes are fragile and break easily, which could limit their practical use in an electrodialysis stack [33].

Regarding the permselectivity to counter-ions, membranes prepared with Amberlite anion exchanger (with or without RE-UF) show the best results in the range of the commercial AMH-PES. Puro-RE-UF and Lew-RE-UF show lower values in comparison with the rest of the membranes. As mentioned before, the inhomogeneity and the presence of cracks in the surface of Puro-RE-UF could cause a reduced permselectivity to counter-ions (see Figure 2b). In the case of Lew-RE-UF, the lower permselectivity could be related to the reduced IEC of this membrane (see Figure 3).

### 3.2. Evaluation of the Selective Ion Transport Properties

**Figure 5:**
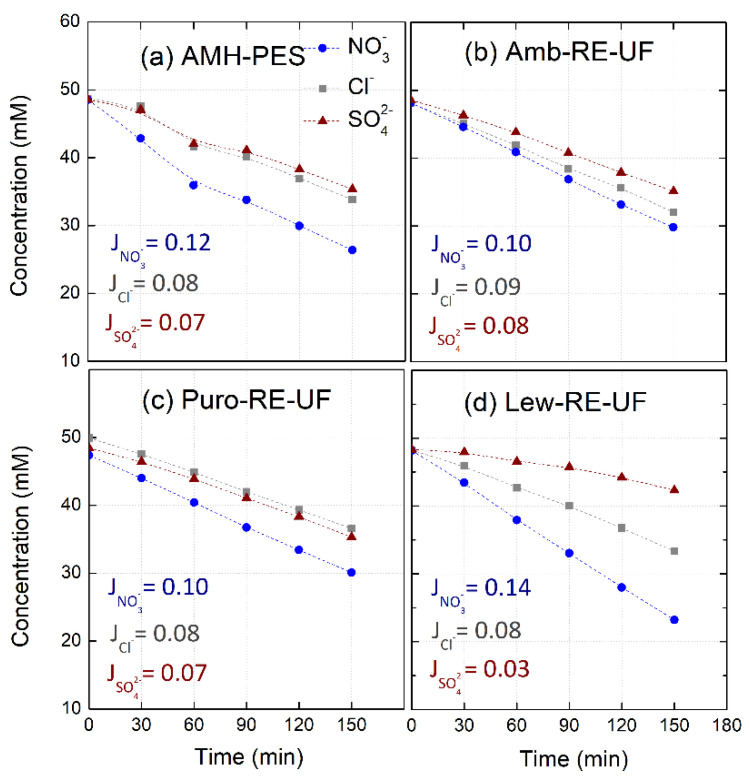
Evolution of anion concentration in the diluted compartment and molar fluxes (*J_i_*, mmol·m^−2^·s^−1^) during electro-separation experiments. The membranes under study were: (**a**) AMH-PES, (**b**) Amb-RE-UF, (**c**) Puro-RE-UF, (**d**) Lew-RE-UF. Feed: NO_3_^−^, Cl^−^ and SO_4_^2−^ (50 mM) added as their sodium salts. *j* = 3.5 mA·cm^−2^.

Figure 5 shows the decreasing concentration of anions in the diluted compartment and their molar fluxes during electro-separation experiments (*j* = 3.5 mA·cm^−2^). The membranes under analysis were AMH-PES and membranes with RE-UF support. Additionally, results corresponding to the experiments performed at *j* = 10 mA·cm^−2^ are reported as Supplementary Materials in Figure S2.

First of all, it can be noticed that nitrates permeate faster with respect to other anions through all the membranes. This effect has been attributed in previous studies to the higher hydration energy of nitrates (see Table 4). Higher hydration energy reduces the amount of water solvation molecules, favoring the interaction between the ion and the functional groups in the membrane and increasing the permeation rate [20,22,45].

Further, the use of different anion exchange resins in membrane preparation strongly affects to molar fluxes of each anion through the membrane. In this sense, Lew-RE-UF membrane could be suitable for ion fractioning, as it achieved a minimized flux of divalent ions ($J_{SO_{4}^{2-}}$ = 0.03 mmol·m^−2^·s^−1^), and a differentiation between the fluxes of monovalent ions ($J_{Cl^{-}}$ = 0.08 mmol·m^−2^·s^−1^ and $J_{NO_{3}^{-}}$ = 0.14 mmol·m^−2^·s^−1^). The differences in the ionic molar fluxes will be reflected in their transport numbers and, consequently, in the permselectivity between the counter-ions (relative transport number) and in the separation efficiency. The performance in ion fractioning of Lew-RE-UF could be related to the hydrophobic propyl chains in the quaternary amine in Lewatit anion exchanger. The presence of hydrophobic alkyl chains in the ion exchange group increases the repulsion of highly hydrated ions (i.e., sulfates) and enhances the transport of less solvated ions (i.e., nitrates) [20,21,22,45,54].

**Figure 6:**
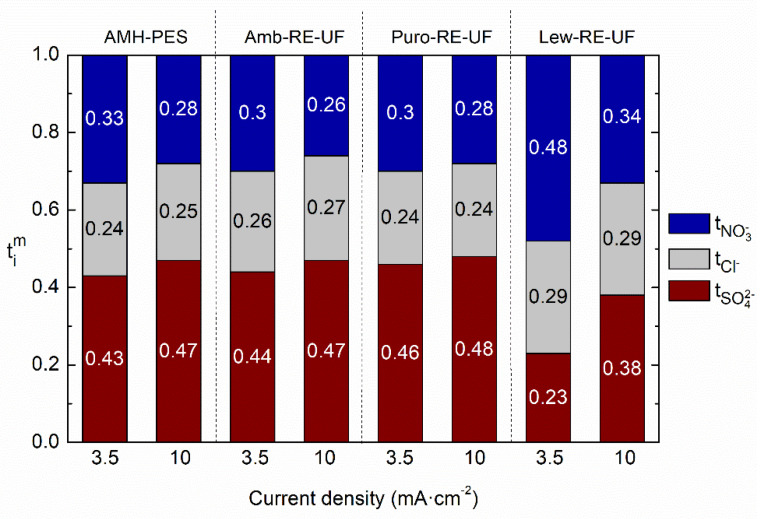
Ion transport numbers ($t_{i}^{m}$) of the counter-ions in the membrane in relation to the operating current (*j* = 3.5 and 10 mA·cm^−2^). Membranes: AMH-PES, Amb-RE-UF, Puro-RE-UF, Lew-RE-UF.

The ion transport number measures the amount of charge that is carried by each counter-ion through the membrane phase. Thus, a faster permeation of nitrate will be reflected in a higher transport number. In this study, the effect of the operating current density in the transport numbers was investigated. Figure 6 shows the differences in ion transport numbers in the experiments conducted at 3.5 and 10 mA·cm^−2^, using AMH-PES and the membranes with RE-UF support. Complementarily, the results for the membranes without mechanical support are shown in Figure S3 (see Supplementary Materials).

**Figure 7:**
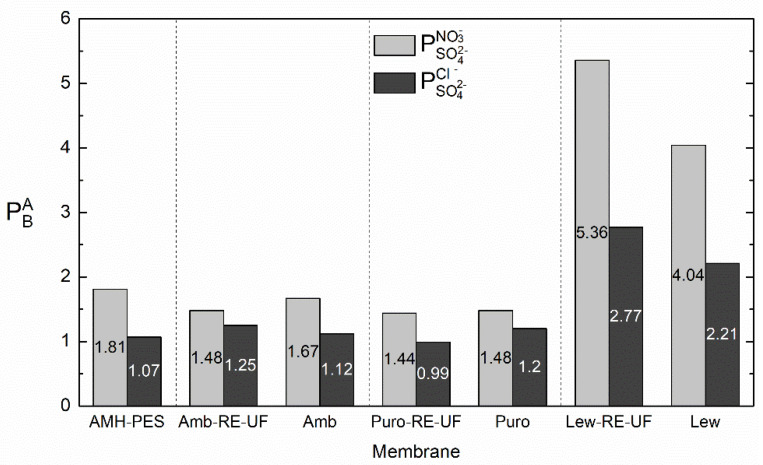
Differences in the permselectivity between the counter-ions ($P_{SO_{4}^{2-}}^{NO_{3}^{-}}$ and $P_{SO_{4}^{2-}}^{Cl^{-}})$ with and without the recycled membrane support (*j* = 3.5 mA·cm^−2^).

It can be observed that operating at relatively low current density (3.5 mA·cm^−2^ instead of 10 mA·cm^−2^) facilitates the transport of monovalent ions (nitrates and chlorides) in detriment of divalent ones (sulfates) in all the tested membranes. The effect is greater in Lew-RE-UF, where the transport number of sulfates is decreased from 0.38 to 0.23 and the transport number of nitrates enhanced from 0.34 to 0.48 when lowering the operational current density. In addition, the differentiation between the transport numbers of chloride and nitrate in Lew-RE-UF (*j* = 3.5 mA·cm^−2^) could result in an efficient separation between the monovalent anions. Interestingly, the employed current density did not significantly affect the transport numbers of chloride in any membrane. The decreased capacity for ion fractioning when operating at high current density could be associated with a decreased Donnan exclusion effect as a result of the saturation of the fixed charged groups in the membrane by the presence of a high concentration of counter-ions in the membrane phase [55]. Further, the higher apparent activation energy of multivalent ions could require a larger amount of electric charge to overcome the energetic barrier and to be transported, avoiding, to some extent, their transport when relatively low current densities are used. [27,28,29]. By the comparison of Figure 6 and Figure S3 (in Supplementary Materials), it can be noticed that the use of the RE-UF support enhanced the transport numbers of nitrate from 0.42 to 0.48 while reduced the transport number of sulfate from 0.29 to 0.23 (data from Lew and Lew-RE-UF, respectively, when *j* = 3.5 mA·cm^−2^). Thus, indicating a positive effect in ion fractionation of the use of the RE-UF membrane. In this line, Figure 7 further analyses the effect of the RE-UF support in permselectivity between counter-ions (or relative transport number). In Figure 7, the results corresponding to the experiments conducted at *j* = 3.5 mA cm^−2^ are presented, while the results of the experiment conducted at 10 mA cm^−2^ are reported as Supplementary Materials in Figure S4.

It can be observed that, in concordance with the transport numbers (Figure 6 and Figure S3), the use of the recycled membrane as support increased the permselectivity between monovalent and multivalent ions in the case of membranes containing Lewatit anion exchanger. In numbers, $P_{SO_{4}^{2-}}^{NO_{3}^{-}}$ was increased from 4.04 to 5.36 and $P_{SO_{4}^{2-}}^{Cl^{-}}$ from 2.21 to 2.77 due to the use of RE-UF support. We first attributed this result to an apparent sieving effect achieved by the use of the RE-UF combined with the casting solution. However, it should be noticed that the positive effect in permselectivity associated with the use of RE-UF membrane was only achieved in membranes containing Lewatit resin. Hence, to obtain selective heterogeneous membranes, the selection of the anion exchange resin is of primary importance.

Overall, the use of Lewatit anion exchanger produces membranes with an enhanced transport of nitrates over sulfates. These membranes could be used for the fractionation of monovalent and divalent anions (i.e., nitrate and sulfate, chloride and sulfate) and for the separation between monovalent anions (i.e., nitrate and chloride), which could facilitate the purification of water for drinking purposes. The efficiency in ion fractionation is increased when the separation is performed at low current density, and the membrane is prepared using a recycled pressure filtration membrane as support. Further, the separation efficiency should be considered in order to define the optimum duration of the separation process.

This work shows an upcycling alternative for end-of-life reverse osmosis membranes by using them as support in an ion-exchange membrane preparation. By means of this, the separation efficiency of the resulting membranes can be upgraded. The use of selective ion exchange resins could be an interesting alternative when target compounds need to be removed from a multi-component mixture. For elucidating the potential application of these membranes, further research on the determination of their economic competitiveness should be conducted. As part of the circular economy approach, these membranes could be tested for nitrate recovery from wastewaters, closing the loops of waste recovery in water purification systems [56] even though the high electrical resistance of the membranes could make them more suitable for passive transport processes such as Donnan dialysis. In parallel, the adequation of using another type of discarded membranes as support could be studied (for instance, discarded ultrafiltration or nanofiltration membranes). Overall, this work is a first attempt at producing nitrate selective membranes by upcycling discarded reverse osmosis membranes.

## 4. Conclusions

This work shows a simple method for the preparation of anion exchange membranes with nitrate selective transport properties. The primary remarks of the present study are:

- Anion exchange membranes were prepared by casting method using a recycled pressure filtration membrane (RE-UF) as support. Homogeneous distribution of the ionic resin on the membrane surface was obtained. Despite differences in anion exchange resin distribution across the membrane section were found;
- The use of an anion exchanger to strengthen hydrophobicity in the functional groups increased the transport of less solvated ions (i.e., nitrates), while highly hydrated ions were repulsed by hydrophobic forces (i.e., sulfates);
- The use of a relatively low current density during the experiment further enhanced the transport of ions with lower charge (monovalent);
- The use of a recycled pressure filtration membrane (RE-UF) as support increased the transport number of nitrates while decreased the transport number of sulfates in the case of membranes containing nitrate selective anion exchange resin. Moreover, the use of recycled membranes as support material provided mechanical stability, and it is an attempt to face the waste management challenge of reverse osmosis desalination. In this line, another type of discarded membranes could be tested as mechanical support.

It can be concluded that the type of anion exchange resin used in membrane preparation is of primary importance in the preparation of selective heterogeneous anion exchange membranes.

## Figures and Tables

**Table 1:** Properties of the anion exchange resins used in this study.

Anion Exchange Resin	Amberlite^®^ IRA-402	Purolite^®^ A600/9413	Lewatit^®^ Sybron Ionac^®^ SR-7
Matrix	Styrene–divinyl benzene cross-linked copolymer	Styrene–divinyl benzene cross-linked copolymer	Styrene–divinyl benzene cross-linked copolymer
IEC (equiv.·L^−1^) *	1.2	1.6	0.8
Ion exchange group	R–(CH_3_)_3_ *N*^+^	R–(CH_3_)_3_ *N*^+^	R–(C_3_H_7_)_3_ *N*^+^
Ionic form	Cl^−^	Cl^−^	Cl^−^

* IEC: ion exchange capacity.

**Table 2:** Summary of the analyzed membranes.

Membrane	Anion Exchange Resin	Mechanical Support
Commercial AMH-PES	Unspecified (ion exchange group, R–(CH_3_)_3_*N*^+^)	Polyester
Recycled ultrafiltration-like membrane (RE-UF)	None	Polyester
Amb-RE-UF	Amberlite^®^ IRA-402	RE-UF
Amb		Without support
Puro-RE-UF	Purolite^®^ A600E/9149	RE-UF
Puro		Without support
Lew-RE-UF	Lewatit^®^ Sybron Ionac^®^ SR-7	RE-UF
Lew		Without support

**Table 3:** Chemical composition analysis by EDX: surface casting layer and polyester layer of the membranes with RE-UF.

**Casting Layer**						
	**Amb-RE-UF**		**Puro-RE-UF**		**Lew-RE-UF**	
Element	% weight	% atomic	% weight	% atomic	% weight	% atomic
C	57.63	77.53	51.84	74.00	52.48	72.96
N	0.04	0.05	0.05	0.06	0.23	0.27
O	5.69	5.74	4.58	4.90	7.89	8.23
S	0.60	0.30	0.64	0.34	0.51	0.27
Cl	36.04	16.39	42.90	20.70	38.89	18.27
**Support Layer**						
	**Amb-RE-UF**		**Puro-RE-UF**		**Lew-RE-UF**	
Element	% weight	% atomic	% weight	% atomic	% weight	% atomic
C	74.78	82.34	64.80	71.62	62.52	69.13
N	0.00	0.00	0.00	0.00	0.01	0.01
O	17.81	14.71	33.38	27.67	36.98	30.67
S	4.64	1.92	0.85	0.35	0.24	0.10
Cl	2.77	1.03	0.96	0.36	0.24	0.09

**Table 4:** Ionic radii, hydrated ionic radii and hydration energy of the studied anions [52,53].

Ion	Ionic Radii (Å)	Hydrated Radii (Å)	Hydration Energy (kJ·mol^−1^)
Cl^−^	1.81	3.32	−381
NO_3_^−^	2.64	3.35	−314
SO_4_^2^^−^	2.9	3.79	−1059

## Supplementary Materials

The following are available online at https://www.mdpi.com/2077-0375/10/12/377/s1, Supplementary Materials. Equations (S1)–(S9). Figure S1: Energy dispersive X-ray (EDX) images of (a) polysulfone surface in the RE-UF support, (b) casting layer and (c) support layer in Amb-RE-UF membrane; (d) casting layer and (e) support layer in Puro-RE-UF membrane, (f) casting layer and (g) support layer in Lew-RE-UF membrane. Table S1. Chemical composition analysis by EDX of the polysulfone surface of the RE-UF membrane. Figure S2: Evolution of anion concentration in the diluted compartment and molar fluxes (*J_i_*, mmol·m^−2^·s^−1^) during electro-separation experiments. The membranes under study were: (a) AMH-PES, (b) Amb-RE-UF, (c) Puro-RE-UF, (d) Lew-RE-UF Feed: NO_3_^−^, Cl^−^ and SO_4_^2−^ (50 mM) added as sodium salts. *j* = 10 mA·cm^−2^. Figure S3: Transport numbers ($t_{i}^{m}$) of the counter-ions in the membrane in relation to the operating current (*j* = 3.5 and 10 mA·cm^−2^). Membranes: Amb, Puro and Lew (membranes without mechanical support). Figure S4. Differences in the permselectivity between the counter-ions (permselectivity between nitrate and sulphate ions, $P_{SO_{4}^{2-}}^{NO_{3}^{-}};\;$and permselectivity between chloride and sulphate ions, $P_{SO_{4}^{2-}}^{Cl^{-}}$) with and without the recycled membrane support (j = 10 mA·cm^−2^ Figure S5. Evolution of the separation efficiency (*S*) during the experiments at *j* = 3.5 mA·cm^−2^ in all the tested membranes. (a) Separation efficiency between chloride and nitrate ions ($S_{{\mathrm{NO}}_{3}^{-}}^{{\mathrm{Cl}}^{-}}$); (b) Separation efficiency between nitrate and sulphate ions ($S_{{\mathrm{SO}}_{4}^{2-}}^{{\mathrm{NO}}_{3}^{-}}$); (c) Separation efficiency between chloride and sulphate ions ($S_{{\mathrm{SO}}_{4}^{2-}}^{{\mathrm{Cl}}^{-}}$). Figure S6. Evolution of the separation efficiency (S) during the experiments at *j* = 10 mA·cm^−2^ in all the tested membranes. (a) Separation efficiency between chloride and nitrate ions ($S_{NO_{3}^{-}}^{Cl^{-}}$); (b) Separation efficiency between nitrate and sulphate ions ($S_{SO_{4}^{2-}}^{NO_{3}^{-}}$); (c) Separation efficiency between chloride and sulphate ions ($S_{SO_{4}^{2-}}^{Cl^{-}}$).
