# Supplementary material for: Nitrate-Selective Anion Exchange Membranes Prepared using Discarded Reverse Osmosis Membranes as Support
Source: Membranes (Basel). 2020 Nov 27;10(12):377. doi: 10.3390/membranes10120377 (PMC7760365; doi:10.3390/membranes10120377)
Supplement: Supplementary file 1 [file membranes-10-00377-s001.pdf]

membranes

Supplementary material

Nitrate Selective Anion Exchange Membranes Prepared using Discarded Reverse Osmosis Membranes as Support

Amaia Lejarazu-Larrañaga [1,2,*], Juan Manuel Ortiz [1], Serena Molina [1], Yan Zhao [3] and Eloy García-Calvo [1,2]

[1] IMDEA Water Institute, Avenida Punto Com, 2, 28805 Alcalá de Henares, Madrid, Spain; juanma.ortiz@imdea.org (J.M.O.); serena.molina@imdea.org (S. M.); eloy.garcia@imdea.org (E.G.-C.).
[2] Chemical Engineering Department, University of Alcalá, Ctra. Madrid-Barcelona Km 33.600, 28871 Alcalá de Henares, Madrid, Spain;
[3] Department of Chemical Engineering, Katholieke Universiteit of Leuven (KU Leuven), Celestijnenlaan 200F, B-3001 Leuven, Belgium; yan.zhao@kuleuven.be
* Correspondence: amaia.ortiz@imdea.org; Tel.: +34-918-30-59-62

Received: 9 November 2020; Accepted: 24 November 2020; Published: date

Supplementary Material and Methods:

The separation efficiency S of component A and B was evaluated as [1]:

$$S\ (t) = \frac{\left(\frac{C_B(t)}{C_B(0)}\right) - \left(\frac{C_A(t)}{C_A(0)}\right)}{\left(1 - \frac{C_B(t)}{C_B(0)}\right) + \left(1 - \frac{C_A(t)}{C_A(0)}\right)} 100\% \tag{S1}$$

where C_A and C_B are the concentrations of A and B in the dilute solution. It is assumed that B is the slowest permeating component, so that in the dilute compartment, the relative concentration of B is always larger than the relative concentration of A.

The separation efficiency reflects the relative difference in the transport rate between the components in the solution and it is ranged from 0 (no separation) to 1 (complete separation, i.e. $C_B(t)$=0; component B completely removed from the dilute fraction). As indicated in the literature [1], the separation efficiency is relatively constant as a function of time, after some initial fluctuations, and it is independent of the geometry of the equipment (e.g. size of the membranes), but may be influenced by the applied voltage.

The separation efficiency can be calculated on the base of the change in molar concentration of each ion with time as it was shown in equation S1. Following equations S2 to S8, it can be considered the separation efficiency as function of the ion transport numbers.

If it is considered that $C_B(t)$ and $C_A(t)$ could be theoretically calculated as indicated below,

$$C_B\ (t) = C_B\ (0) - \frac{t_B^m\ j\ A}{|z_B\ |F\ V} \cdot \tag{S2}$$

being t_B^m the transport number of the component B in the membrane phase, j (mA·cm^{-2}) the current density, A (cm^2) membrane effective area, z_i the valence of the ion i, F (C·mol^{-1}) Faraday's constant and V (cm^3) the volume of the dilute compartment . Similarly, the next equation could be used for component A,

$$C_A\ (t) = C_A\ (0) - \frac{t_A^m\ j\ A}{|z_A\ |F\ V} \cdot \tag{S3}$$

In order to simplify equation S1, it is possible to define k as indicated below,

$$k = \frac{j\ A}{F\ V} \cdot \tag{S4}$$

and then, equation S2 could be expressed as,

$$C_B\ (t) = C_B\ (0) - \frac{t_B^m}{|z_B|} k \cdot \tag{S5}$$

Then equation S1 could be written as:

$$S\ (t) = \frac{\left(\frac{C_B(0) - \frac{t_B^m}{|z_B|}k}{C_B(0)}\right) - \left(\frac{C_A(0) - \frac{t_A^m}{|z_A|}k}{C_A(0)}\right)}{\frac{-\frac{t_B^m}{|z_B|}k}{C_B(0)} - \frac{\frac{t_A^m}{|z_A|}k}{C_A(0)}} \cdot 100\% \tag{S6}$$

When the experiment is carried out with equal initial concentrations for both components, i.e. $C_A(0) = C_B(0)$, A and B have the same charge (for example, $|z_B| = |z_A| = 1$), equation S6 is simplified as indicated below,

$$S\ (t) = \frac{t_A^m - t_B^m}{-\left(t_A^m + t_B^m\right)} 100\% \cdot \tag{S7}$$

And thus,

$$S\ (t) = \frac{t_A^m - t_B^m}{t_A^m + t_B^m} \cdot 100\% \tag{S8}$$

In this particular case, ($C_A(0) = C_B(0)$, $|z_B| = |z_A| = 1$), the separation efficiency (S) could be considered as an indirect measurement of the difference between transport numbers for A and B, as reported in [1]. In this way, S = 0 % indicates that $t_A^m = t_B^m$, thus both ions are transported with the same velocity through the membrane (i.e. the ions transport the same amount of electric charge during migration). If the value of S = 50% it is easy to determine using S6 that $t_A = 3\ t_B$, indicating a higher migration/transport of A compared to B through the membrane. Finally, the value of S = 100 % would indicate that all the charge is transported by A (i.e. $t_A^m + t_B^m \approx t_A^m$) and the transport of charge due to B is negligible. Finally, for mixtures of monovalent and multivalent ions, the valence of each ion should be taken into account and thus,

$$S\ (t) = \frac{\frac{t_A^m}{|z_A|} - \frac{t_B^m}{|z_B|}}{\frac{t_A^m}{|z_A|} + \frac{t_B^m}{|z_B|}} \cdot\ 100\% \tag{S9}$$

In this case, S = 0 % indicates that $\frac{t_A^m}{|z_A|} = \frac{t_B^m}{|z_B|}$. If it is considered the case in which $|z_A| = 1$ (monovalent ion) and $|z_B| = 2$ (divalent ion), then $2t_A = t_B$ and thus, the divalent ion is transporting twice the charge of the monovalent ion, being the molar flux of both ions the same. When the value of S = 50 %, it could be calculated using S9 the value of $t_A = \frac{3}{2} t_B$. Again, if S = 100 %, then $t_B^m \approx 0$, and all the charge is transported by component A.

It is important to note that the previous analysis would be accurate as long as i) initial concentrations are equal (i.e. $C_A(0) = C_B(0)$) and ii) and the transport number for each ion could be considered constant (i.e. no significant change of concentration for $C_A(t)$ and $C_B(t)$ during the experiments).

Supplementary Results and Discussion:

Membrane characterization

Figure S1 shows the Energy-dispersive X-ray (EDX) images of the RE-UF membrane (used as support) and the prepared AEMs. In this figure, the elemental composition of the casting layer and the support layer of the AEMs can be observed.

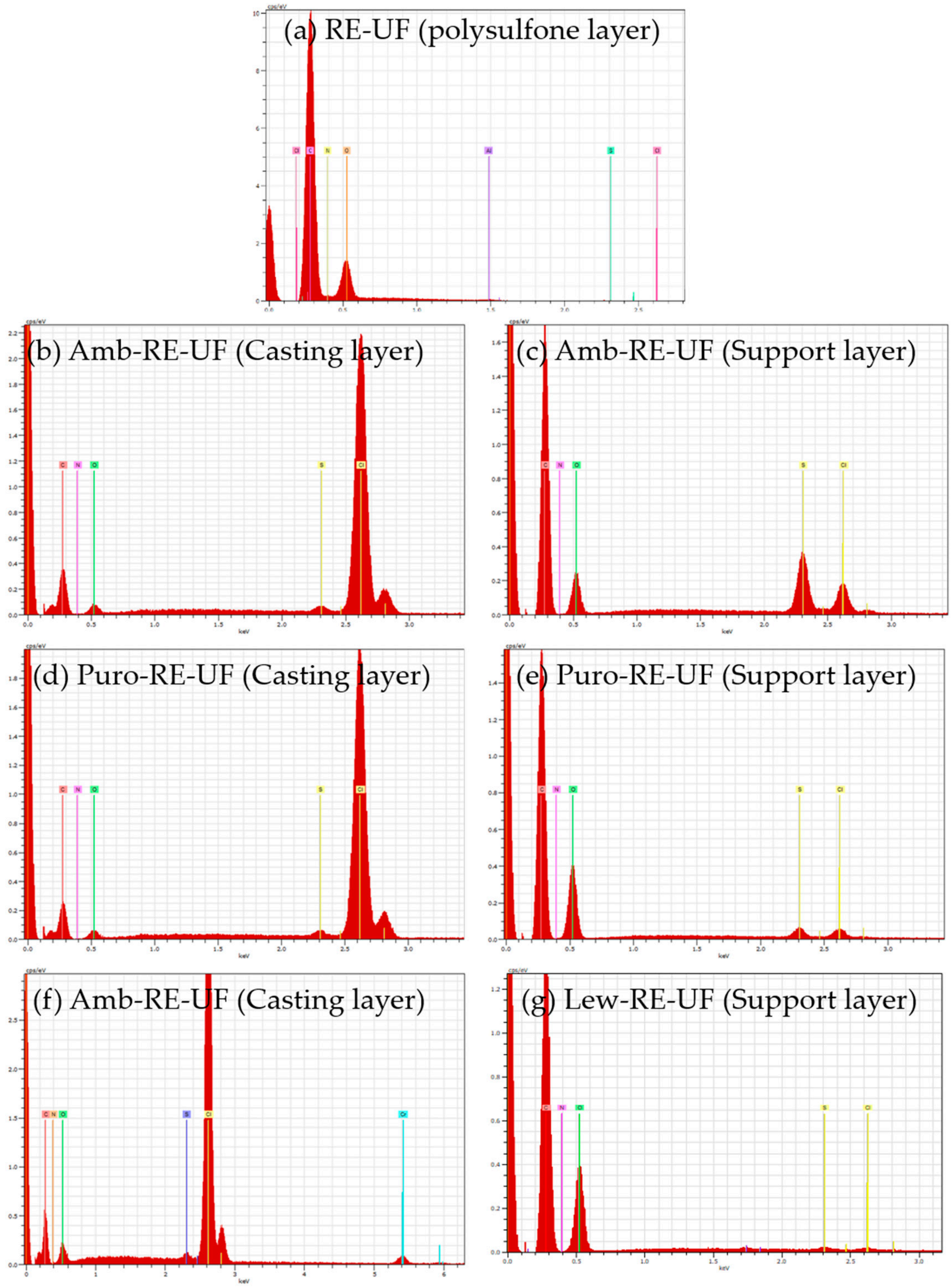

Figure 1. Energy dispersive X-ray (EDX) images of (**a**) polysulfone surface in the RE-UF support, (**b**) casting layer and (**c**) support layer in Amb-RE-UF membrane; (**d**) casting layer and (**e**) support layer in Puro-RE-UF membrane, (**f**) casting layer and (**g**) support layer in Lew-RE-UF membrane.

Table S 1. Chemical composition analysis by EDX of the polysulfone surface of the RE-UF membrane. Polysulfone Surface in RE-UF

Element	% weight	% atomic
C	73.74	78.56
N	4.97	4.54
O	20.95	16.75
Al	0.27	0.13
S	0.07	0.03
Cl	0	0

All the prepared membranes show Cl in their elemental composition, due to the casting of the polymeric solution containing PVC as binder. The presence of Cl was not detected in the surface of the RE-UF membrane, indicating that all the Cl in the prepared membranes corresponds to the PVC in the casting solution. As explained in the manuscript, a peak of Cl was observed in both sides of the prepared membranes (in the casting and the support layers). The peak of Cl in the casting layer is large, due to the dense film of PVC formed in the membrane surface. However, the amount of PVC that totally crossed the RE-UF support was very low as it is reflected in the low signal of Cl in the support layer.

In the prepared AEMs, the peak of N corresponds to the amine group in the anion exchange resin. The low intensity of N peaks confirms that the resin particles are covered by a dense film of PVC. *N* was not found in the support layer which could reflect an unequal distribution of the ion exchange particles across the membrane section (lower concentration of ion exchange particles embedded in the RE-UF support in comparison with the casting layer).

Evaluation of the selective ion transport properties

Figure S2, shows the decreasing concentration of anions in the dilute compartment and molar fluxes during the electro-separation experiments performed at 10 mA·cm^{-2}. The tested membranes were AMH-PES and membranes with recycled support.

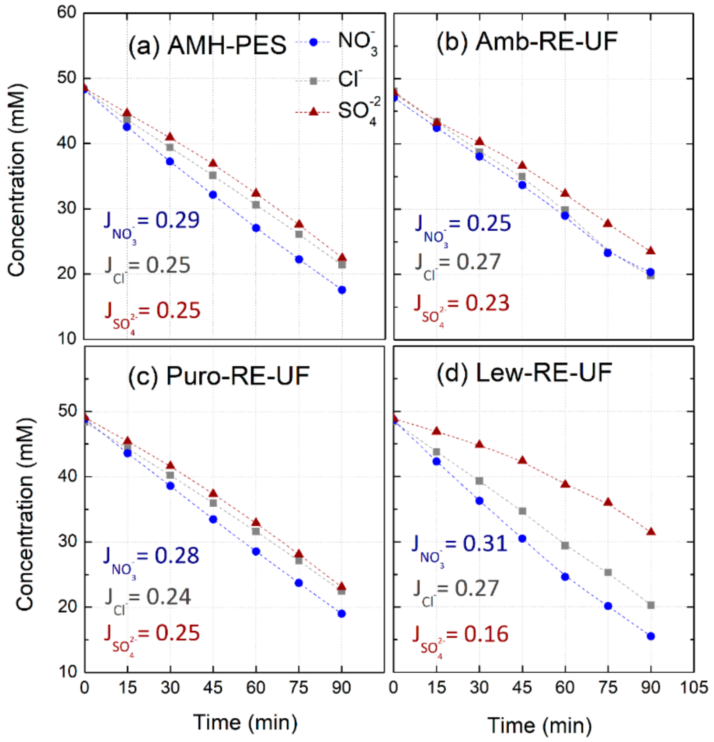

Figure S 1. Evolution of anion concentration in the diluted compartment and molar fluxes (J_i, mmol·m^{-2}·s^{-1}) during electro-separation experiments. The membranes under study were: (**a**) AMH-PES, (**b**) Amb-RE-UF, (**c**) Puro-RE-UF, (**d**) Lew-RE-UF. Feed: NO_3^-, Cl^- and SO_4^{2-}(50 mM) added as sodium salts. *j*= 10 mA·cm^{-2}.

It can be observed that the molar fluxes are increased in respect to figure 5 (in the manuscript) due to the increase of the current density. As explained in the manuscript, in all the tested membranes, the permeation of nitrate is faster than the fluxes of chlorides and sulfates which can be related to the higher hydration energy of nitrates. In addition, the capacity for achieving an effective ion fractionation relies upon the anion exchanger used in membrane preparation. In this manner, the use of Lewatit anion exchanger declines the flux of sulfates while increases the flux of nitrates. This effect could be attributed to the hydrophobic propyl chains in the functional group of the Lewatit anion exchanger.

Figure S3 shows the differences in transport properties of the membranes without mechanical support when conducting the experiment at 3.5 and 10 mA·cm^{-2}.

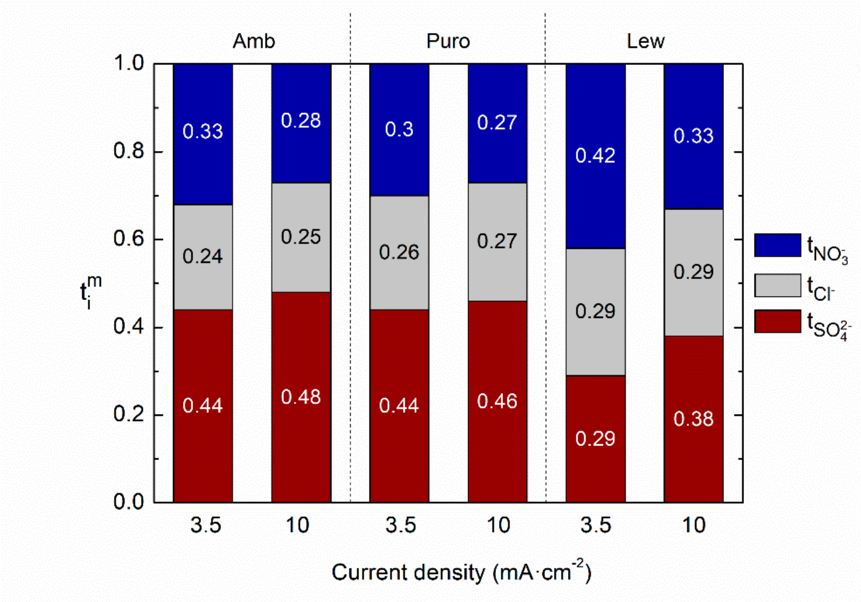

Figure S 2. Transport numbers (t_i^m) of the counter-ions in the membrane in relation with the operating current (*j*= 3.5 and 10 mA·cm^{-2}). Membranes: Amb, Puro and Lew (membranes without mechanical support).

It can be observed that, in concordance with the manuscript, conducting the experiments at 3.5 mA·cm^{-2} results in a larger transport of nitrates in detriment of sulfates. The effect is greater in the case of Lew membrane where the transport number of sulfate is reduced from 0.38 to 0.29 while the transport number of nitrates is enhanced from 0.33 to 0.42 when lowering the operating current density (from 10 to 3.5 mA·cm^{-2}). As mentioned in the manuscript, the decrease in the rejection of sulfate at 10 mA·cm^{-2} can be attributed to a decreased Donnan exclusion effect. While at 3.5 mA·cm^{-2} the higher apparent activation energy of sulfates could reduce its transportation through the membrane. In addition, it can be observed a great differentiation between the transport numbers of nitrate and chloride in Lew membrane, further increased when the experiment is operated at low current density ($t_{NO_3^-}$ 0.42 and t_{Cl^-} 0.29).

Figure S4 shows the permselectivity between counter-ions (relative transport number) in respect to sulfates, achieved by the membranes when operating at 10 mA·cm^{-2}. In this figure, the effect of the mechanical support in the permselectivity between counter-ions can be appreciated.

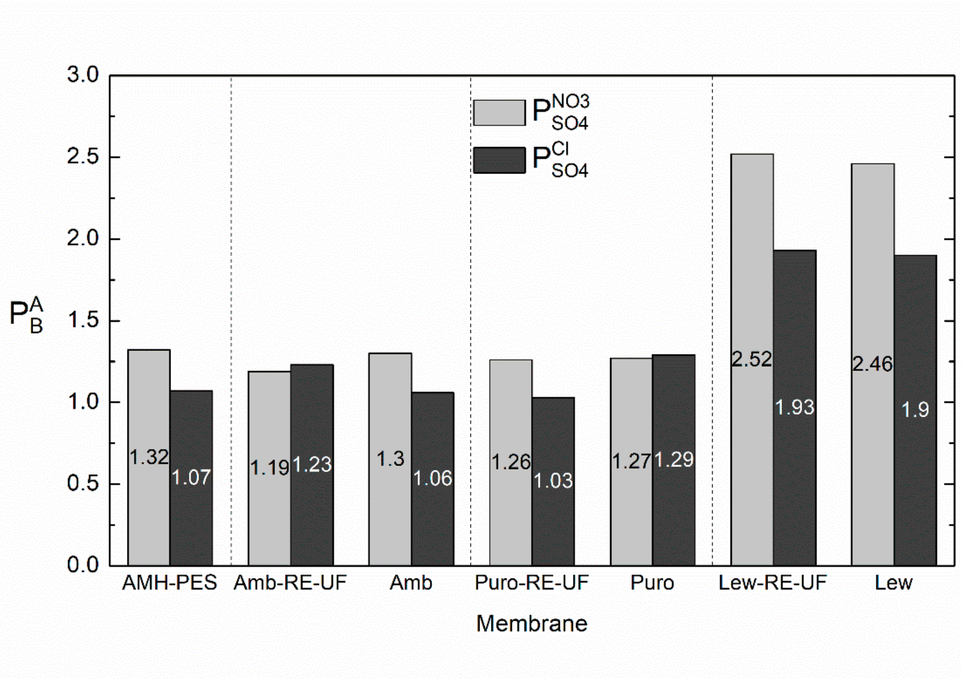

Figure S 3. Differences in the permselectivity between the counter-ions (permselectivity between nitrate and sulphate ions, $P_{SO_4^{2-}}^{NO_3^-}$; and permselectivity between chloride and sulphate ions, $P_{SO_4^{2-}}^{Cl^-}$)with and without the recycled membrane support (j= 10 mA·cm^{-2}).

It can be observed that, in comparison with figure 7 (in the manuscript), the permselectivity was decreased in all the cases due to operating at 10 mA·cm^{-2}. In the case of membranes containing Lewatit anion exchanger, the use of the RE-UF membrane as mechanical support slightly enhanced the permselectivity to monovalent ions. However, the main differences in permselectivity between counter ions rely on the type of anion exchanger used in membrane preparation and in the use of an adequate operating current during the separation experiments.

Another parameter for analyzing the differences in the transport properties between the ions is the separation efficiency (*S*). Figure S5 and S6 show the evolution of the separation efficiency during the experiments performed at 3.5 and 10 mA·cm^{-2}, respectively.

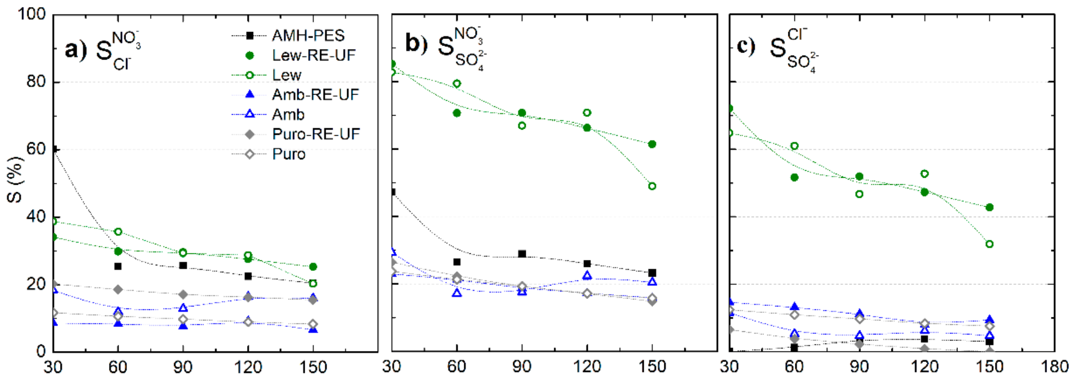

Figure S 4. Evolution of the separation efficiency (S) during the experiments at *j* = 3.5 mA·cm^{-2} in all the tested membranes. (**a**) Separation efficiency between chloride and nitrate ions ($S_{NO_3^-}^{Cl^-}$); (**b**) Separation efficiency between nitrate and sulphate ions ($S_{SO_4^{2-}}^{NO_3^-}$); (**c**) Separation efficiency between chloride and sulphate ions ($S_{SO_4^{2-}}^{Cl^-}$).

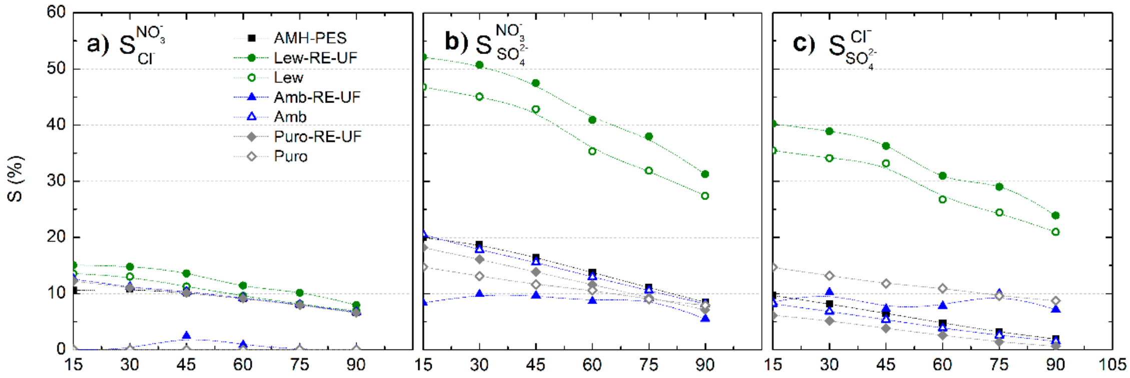

Figure S 5. Evolution of the separation efficiency (S) during the experiments at j = 10 mA·cm-2 in all the tested membranes. (**a**) Separation efficiency between chloride and nitrate ions ($S_{NO_3^-}^{Cl^-}$); (**b**) Separation efficiency between nitrate and sulphate ions ($S_{SO_4^{2-}}^{NO_3^-}$); (**c**) Separation efficiency between chloride and sulphate ions ($S_{SO_4^{2-}}^{Cl^-}$).

As mentioned before, *S* reflects differences in the transport between the components A and B. In a general sense, a large difference between the transport numbers of A and B is reflected in higher separation efficiency (see equations S2 and S9). As a consequence, *S* is in concordance with previously explained results ($S_{SO_4^-}^{NO_3^-} > S_{SO_4^{2-}}^{Cl^-} > S_{Cl^-}^{NO_3^-}$). Again, Lew-RE-UF membrane show the highest separation efficiency, achieving $S_{SO_4^-}^{NO_3^-}$ = 85%, $S_{SO_4^{2-}}^{Cl^-}$= 72% and $S_{Cl^-}^{NO_3^-}$ = 34% in the beginning of the experiment (30 min) when it is conducted at low current density (*j*=3.5 mA·cm-2) (Figure S5). The separation efficiency was considerably decreased in the experiments conducted at 10 mA·cm-2, and in this case, Lew-RE-UF achieved $S_{SO_4^{2-}}^{NO_3^-}$= 52%, $S_{SO_4^{2-}}^{Cl^-}$= 40% and $S_{Cl^-}^{NO_3^-}$=15% in the beginning of the experiment (15 min) (Figure S6).

This parameter further demonstrated the feasibility of using Lew-.RE-UF membrane for ion fractioning. In the case of Amb-RE-UF and Puro-RE-UF, the separation efficiency is almost constant during the experiment as long as the concentrations of the counter-ions are maintained in an equimolar relation. However, in the case of the membranes prepared with Lewatit anion exchanger, (Lew-RE-UF and Lew), the separation efficiency shows a decreasing tendency during the experiment. These results can be attributed to differences in the permeation rates of nitrate, chloride, nitrate and sulfate, which results in a faster depletion of nitrates, followed by chlorides, in the dilute compartment. When the concentration of nitrate and chloride in the dilute compartment is too low to transport the electric charge that is being applied to the system, then sulfates will be transported through the membrane, decreasing the separation efficiency as it is observed in figures S5 and S6. Overall, S is a parameter that can be useful to define the optimum duration of the separation process for maintaining of high separation efficiency.

References

1. Van der Bruggen, B.; Koninckx, A.; Vandecasteele, C. Separation of monovalent and divalent ions from aqueous solution by electrodialysis and nanofiltration. *Water Res.* **2004**, *38*, 1347–1353, doi:10.1016/j.watres.2003.11.008.
